# Anaphylactic reaction to platelet transfusion as the initial symptom of an undiagnosed systemic mastocytosis: a case report and review of the literature

**DOI:** 10.1186/1752-1947-8-389

**Published:** 2014-11-26

**Authors:** Clifford R Blieden, German Campuzano-Zuluaga, Adrienne Moul, Jennifer R Chapman, Maureen Cioffi-Lavina, Offiong F Ikpatt, Gerald E Byrne, Francisco Vega

**Affiliations:** 6 Department of Pathology and Laboratory Medicine, University of Miami Miller School of Medicine/Sylvester Comprehensive Cancer Center in affiliation with Jackson Memorial Hospital, Miami, FL USA; 7 Department of Pathology and Laboratory Medicine, University of Miami Miller School of Medicine/Jackson Memorial Hospital, University of Miami Hospital, Suite 4070, 1400 NW 12th Ave, Miami, FL 33136 USA

**Keywords:** Anaphylaxis, Platelet transfusion, Systemic mastocytosis, Transfusion reaction

## Abstract

**Introduction:**

The association between anaphylactic reactions and systemic mastocytosis is well documented. However, platelet transfusion has not previously been reported as a potential elicitor of anaphylaxis in the context of systemic mastocytosis.

**Case presentation:**

We describe the clinicopathological findings of a 59-year-old Latin American man who presented to the emergency room with fatigue, leukocytosis, thrombocytopenia and mild hepatosplenomegaly. He developed two separate, temporally associated and severe anaphylactic reactions after receiving platelet transfusions. The result of a laboratory investigation for clerical errors and Coombs test was negative. Pre- and post-transfusion urine samples were negative for hemolysis. Bone marrow biopsy and aspirate smears performed demonstrated involvement by systemic mastocytosis, which had been previously undiagnosed.

**Conclusions:**

We posit the transfusion reaction to be an anaphylactic reaction to transfused products as a result of heightened allergic sensitivity due to the underlying systemic mastocytosis. To the best of our knowledge, this is the first reported case of a severe anaphylactic-type reaction to blood products occurring in the setting of a previously undiagnosed systemic mastocytosis. Furthermore, it seems there are no published studies closely examining the relationship between hematopoietic neoplasms and transfusion reactions in general.

## Introduction

Mast cells were first described by Paul Erlich in 1878 and play an important role in allergy, anaphylaxis, and defense against pathogens. These cells have abundant cytoplasmic granules which are composed predominantly of heparin and histamine [1]. Normal degranulation of mast cells occurs via antigen-immunoglobulin (Ig) E complex-mediated binding to membrane-bound FcϵRI (high-affinity IgE) receptors which in turn release histamine and heparin-rich granules into the circulation [1].

Non-IgE-mediated mechanisms of mast cell degranulation can also occur. These include predominantly physical stimuli such as cold temperature, radiation, ethanol, exercise, and friction in addition to several exogenous substances such as medication, radio-contrast media and venom [2]. Several blood-derived substances may also initiate the process of mast cell degranulation, including immune aggregates, Igs, platelets and T cells [2].

Anaphylaxis is the result of massive mast cell activation and degranulation and is a serious and life-threatening allergic reaction that occurs with rapid onset [2]. Symptoms include but are not limited to hives, angioedema, wheezing, and hypotension with tachycardia. Common triggers include food allergies, insect bites, and chemical exposures among other things. However, etiologic agents are frequently difficult to identify in clinical practices. The estimated prevalence of people having had at least one anaphylactic reaction is 0.5 to 2% and is thought to be increasing [3].

Systemic mastocytosis (SM) is part of a spectrum of diseases which are characterized by the proliferation of neoplastic mast cells [4]. Clinical symptoms of SM vary in severity and can occur over a broad range of organ systems, making the clinical diagnosis difficult. Common symptoms include constitutional symptoms, skin manifestations, and musculoskeletal symptoms all of which are termed “mast cell mediator-related systemic symptoms”. SM tends to present after the second decade of life, whereas the more common cutaneous mastocytosis has a predilection towards pediatric populations [4]. Clinical management of mastocytosis ranges from palliative means in mild cases to cladribine in more severe cases. SM can be associated with *KIT* mutations. In fact, imatinib has a role in severe diseases with *KIT* gene mutation, particularly in those with associated clonal myeloid neoplasms. Furthermore, *KIT* mutations confer a resistance to imatinib therapy and a poorer prognosis in severe cases [5].

Anaphylaxis is included in the category of mast cell mediator-related symptoms and is not uncommonly seen in patients with SM [6–8]. A recent Swedish study of 84 adult patients with SM revealed that 36% of these patients had at least one episode of anaphylaxis [9]. An American study of 120 adult and pediatric patients found a 49% incidence of anaphylaxis in patients with SM [10]. As is commonly the case in other patients with anaphylaxis, a causative trigger was not identified in the majority of these cases.

We here present a case of SM which was diagnosed after two sequential episodes of anaphylaxis, each occurring with platelet transfusion as the precipitating event. This report serves as a reminder to clinicians of the possibility of an underlying SM in the settings of an anaphylactic reaction to blood products as well as the clinical implications of the use of transfused blood products in such patients. Finally, this study highlights the need for further studies investigating the association between transfusion reactions and hematopoietic neoplasms in general, a virtually unexplored topic of clinical interest.

## Case presentation

A 59-year-old Latin American man with a reported history of atrial fibrillation presented to the emergency room with fatigue, progressive abdominal pain, and weight loss. Significant laboratory findings were elevated white blood count (37×10^3^/μL) with markedly increased eosinophilia (46% of manual leukocyte differential cell count) and thrombocytopenia (17×10^3^/μL). Hemoglobin was 11.5g/dL and hematocrit was 34.3%. His physical examination was notable for mild hepatosplenomegaly. An initial bone marrow biopsy performed was non-diagnostic due to inadequate material.

He was admitted for unexplained leukocytosis and thrombocytopenia. On admission, he received single donor platelet transfusion. This was performed in part because despite the lack of active bleeding, he had areas of petechiae on the upper extremities and hard palate and gave a reported history of melena. Almost immediately after initiation of platelet transfusion (per nursing notes less than 10 minutes after beginning transfusion) he developed hypotension (blood pressure 77/40mmHg), diaphoresis, respiratory distress, and atrial fibrillation with rapid ventricular response (heart rate 200 beats per minute). He was urgently treated with amiodarone, metoprolol, intravenous diphenhydramine, and 1500mL normal saline bolus. He did not have urticarial symptoms, nor did he report wheezing per se, however, he did complain of shortness of breath. Mild pulmonary edema was noticed on the subsequent chest X-ray; however, this study was not performed in the immediate post-episodic interval, and in fact was performed 6.5 hours after the episode. No fever occurred. He had not been taking angiotensin-converting enzyme inhibitors. Cardiac enzymes were negative at the time and remained negative in the days following the episode. Brain natriuretic peptide levels were not ordered. With prompt medical attention, he was quickly stabilized and subsequently transferred to the intensive care unit.

The transfusion medicine service was consulted for investigation of the cause of the transfusion reaction and guidance for the safety of future blood product transfusions. Clerical errors were ruled out by standard laboratory protocol. Both direct and indirect Coombs tests were negative. Pre- and post-transfusion urine samples did not demonstrate hemolysis. The initial interpretation was that the symptoms were possibly the result of either a transfusion-related acute lung injury (TRALI) or an anaphylactic reaction. The lack of urticarial symptoms made for ambiguity in diagnosing an anaphylactic reaction; however, the loss of hemodynamic stability, possibility due to vasodilation, kept anaphylaxis in the differential. TRALI was a consideration because he had complained of shortness of breath. The lack of an immediate post-interval chest X-ray confounded the exclusion of TRALI, which has a characteristic marked transient pulmonary edema. Although the etiology of the reaction was unclear at this point, a TRALI was favored over an allergic-type anaphylactic reaction. Concurrently, a bone marrow biopsy had been performed but was unsatisfactory and thus non-diagnostic.

He was stabilized and transferred to the floor unit. With the diagnosis of an allergic-type reaction not fully elucidated, and the diagnosis of SM not yet made or even suspected, another platelet transfusion with close medical attention was attempted for the same clinical reasons at presentation. An almost identical reaction occurred. This time, he had received pretreatment with intravenous diphenhydramine and Solu-Medrol® (methylprednisolone sodium succinate). Within a short period of time after initiation of platelet transfusion (approximately 15 minutes after initiation of transfusion), he experienced shortness of breath, hypotension (blood pressure 73/42mmHg), and symptomatic cardiac arrhythmias. Identical medical interventions using antiarrhythmics, beta-blockers, and antihistamines were again implemented. He responded quickly to medical intervention and was subsequently again transferred to the intensive care unit. A second bone marrow examination was performed and a diagnosis of SM was made.

**Figure 1:**
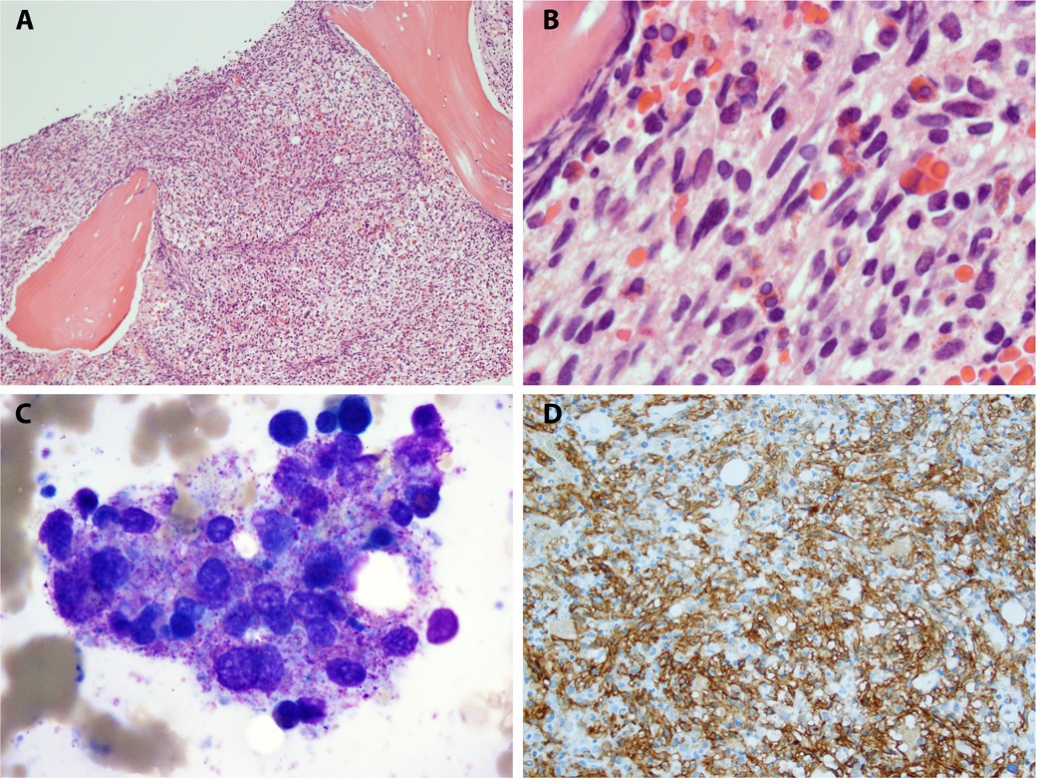
**Bone marrow involvement by mastocytosis. A**. The bone marrow core biopsy is hypercellular (90%) due to the presence of a proliferation of spindle cells admixed with small lymphocytes and eosinophils. **B**. High power image of mast cell aggregates in bone marrow core biopsy demonstrating marked spindle cell morphology. **C**. Clusters of hypogranular mast cells were seen in the bone marrow aspirate smears. **D**. The neoplastic mast cells were positive for CD117 and CD25 (shown).

Peripheral blood demonstrated a leukocytosis with significant eosinophilia, with 49% of nucleated cells being eosinophils. All leukocytes were morphologically unremarkable. Circulating blasts were not seen. His bone marrow examination was significant for hypercellularity with marked spindle cell proliferation constituting approximately 90% of the marrow cellularity. Bone marrow aspirate had few particles; however, cellular material present consisted predominantly of clustered mast cells (Figures 1A to 1C). Immunohistochemical studies demonstrated spindle cells to be positive for CD25 (Figure 1D) in addition to CD2, CD117, and tryptase. Based on these findings, a diagnosis of SM was made. Molecular studies showed a *KIT* gene mutation D816V by polymerase chain reaction. Results from serum tryptase levels collected after the first episode were made available to the clinicians and were found to be abnormally elevated (>200ng/mL; reference range 5 to 10ng/mL). Ig levels performed after transfusion were essentially normal for IgG, IgA, and IgE. IgM was found to be slightly elevated (234mg/dL; reference range 40 to 230mg/dL). He was unsure about his prior transfusion history, and records from outside institutions were unavailable. This patient was lost to follow up for clinical purposes, however our institution was notified of his death approximately 4 months after his initial diagnosis.

Because of the unlikelihood of having two successive TRALI reactions in addition to the confirmed diagnosis of SM, the etiology of the transfusion reactions was thought to be a result of induced mast cell hypersensitivity; either by the platelets themselves (i.e. membrane phospholipids), concentrated platelet derived substances contained in the transfused units (serotonin, histamine, etc.), or both. The fact that he had a virtually identical reaction to another attempted platelet transfusion from a different donor virtually excluded TRALI as a possibility.

## Conclusions

Based on clinical grounds and pathological findings, we infer that the most likely etiology of this patient’s transfusion reaction was anaphylactic reaction as a result of hypersensitivity due to SM. Because of this diagnosis, the clinical team opted to manage the patient such that future transfusions would be performed conservatively in acute care settings, with washed blood products, and using pretreatment with corticosteroids and antihistamines. As in all allergic-type reactions, IgA was considered a possibility; however, studies collected on the patient after attempted transfusion were within normal limits. We acknowledge that, in general, transfusion may alter IgA levels and give a falsely normal reading. This is a more common phenomenon in plasma transfusion [11]. Considering that the product transfused was platelets as opposed to plasma, and that such a limited amount was transfused, our impression is that the measured normal IgA levels were probably not altered by the transfused platelets and that the laboratory and clinical findings did not support the diagnosis of IgA deficiency.

Anaphylactic reactions are a known entity among transfusion reactions, with an incidence of approximately 1:20,000 to 1:50,000 [11]. Symptoms are not unlike anaphylactic symptoms in other circumstances and may include hypotension, tachycardia, respiratory difficulty, urticaria, and other allergic-type symptoms. Patients who have an anaphylactic reaction to transfused blood products are subsequently managed with washed blood products as needed in addition to pretreatment with antihistamines and corticosteroids as deemed necessary. The clinical team was counseled in this regard and urged only to transfuse with acute care immediately available. The clinical team never again attempted transfusion over the course of this patient’s hospitalization. A common cause for anaphylactic reaction is transfusion of products to patients with IgA deficiency, who subsequently react to the foreign IgA antigen in the donor unit [11]. As previously mentioned, this patient had normal serum IgA levels. He was subsequently lost to follow up and his clinical course after discharge remains unknown.

It is not uncommon for transfusion reactions to be difficult to classify as many do not fit perfectly into the criteria established for various entities. This is particularly true when TRALI is included in the differential as was initially the case in this scenario. The reported incidence of TRALI ranges from 1:1200 to 1:190,000 [11]. This variance may be because of the frequent difficulty in identifying an unequivocal TRALI. The mechanism of TRALI is thought to be due to human leukocyte antigen (HLA) antibodies of the donor unit directed against the recipient, which classically manifests as a dramatic transient pulmonary edema shortly after transfusion [11]. Blood units with a higher incidence of HLA-associated donor-related antibodies, which increase the risk of TRALI, have been found to have an increased incidence in multiparous female donors [11].

In this particular case, the chest X-ray was not taken in the immediately post-episodic period, and hence the findings of minimal pulmonary edema confounded the potential exclusion of TRALI as a cause of this patient’s symptoms. Cardiac symptoms are not included in the definition of TRALI. This patient’s history of cardiac disease further confounded the exclusion of TRALI diagnosis and also made for difficulty in attributing the hypotension to anaphylaxis. That is to say, it was thought that an exacerbation of the pre-existing cardiac disease by a suspected TRALI might have given rise to the hypotension. The fact that he had the exact reaction to another platelet transfusion virtually excluded TRALI as a possibility. Furthermore, the subsequent bone marrow findings identifying an undiagnosed mastocytosis led to the conclusion that the most likely etiology of this patient’s reaction was anaphylactic hypersensitivity.

The association of an anaphylactic reaction to platelet transfusion arising in the setting of an underlying SM, as seen in this case, has not previously been reported in the literature (PubMed). In fact, it appears that the relationship between transfusion reactions and hematopoietic neoplasms as a whole has yet to be explored as a topic of clinical interest. As previously mentioned, the clinical manifestations of mastocytosis are broad ranging. This in combination with the fact that the disease is a relatively uncommon entity makes the diagnosis easily missed by clinicians.

We suspect that the mechanism of reactions seen in this case is part of a mast cell activation syndrome occurring in the setting of SM [6]. It is possible that this was an IgE-mediated allergic reaction and subsequent massive mast cell degranulation, although this seems unlikely given that he had normal IgE levels. Another possible mechanism is the massive activation of neoplastic mast cells by leukocyte or platelet-derived substances contained in the transfused units. Some possible candidates include platelet-activating factor (PAF) and stem cell factor (SCF). Both PAF and SCF are found in significant amounts in transfused platelets. Notably, PAF has been implicated in the development of anaphylactic reactions via histamine release [7]. Also, SCF is known to not only stimulate proliferation of mast cells via c-kit but also to be a potent mast cell agonist leading to histamine release [12–14]. It might also be true that the activating *KIT* gene mutation present in SM may have played a role in this patient’s adverse reaction due to increased susceptibility to other activating stimuli; however, this is only conjecture. In any regard, the exact mechanism of mast cell activation cannot be determined with certainty in this case. Understanding the exact mechanism of anaphylaxis would require an experimental study involving an animal model.

Further identification and understanding of the association between SM and potential anaphylactic-type transfusion reactions is important because the awareness of this association may allow for more optimal management in patients with SM. Specifically, clinicians should be aware that patients with SM who require transfusion of blood products may require pretreatment with antihistamines and corticosteroids. This is an important topic of consideration because the physiological burden of SM in the marrow leads to marrow suppression and subsequent need for transfusion. Furthermore, this case serves as an example of the need for investigation of the potential correlation between hematopoietic neoplasms and transfusion reactions. Although this topic has yet to be explored, the connection between the two entities seems intuitively possible. Because of the complications that may arise with transfusion of blood products, one would think that such research could be of great clinical importance as patients with hematopoietic neoplasms are likely to require blood product transfusions over the course of their disease.

## Consent

Institutional Review Board approval (IRB 20140281) was received for this manuscript as we were unable to contact the deceased patient’s next of kin for consent to publish despite all reasonable attempts. All efforts were made to keep our deceased patient's identity anonymous and there is no reason to believe that our patient would have objected to publication of this case report.

## Abbreviations

HLA: Human leukocyte antigen
Ig: Immunoglobulin
PAF: Platelet-activating factor
SCF: Stem cell factor
SM: Systemic mastocytosis
TRALI: Transfusion-related acute lung injury.
